# Combining self-management cues with incentives to promote interdental cleaning among Indian periodontal disease outpatients

**DOI:** 10.1186/s12903-016-0164-5

**Published:** 2016-01-28

**Authors:** Pempa Lhakhang, Kyra Hamilton, Nayantara Sud, Shonali Sud, Jeroen Kroon, Nina Knoll, Ralf Schwarzer

**Affiliations:** Department of Psychology, Freie Universität Berlin, Berlin, Germany; Menzies Health Institute Queensland and School of Applied Psychology, Griffith University, Brisbane, QLD Australia; School of Psychology and Speech Pathology, Curtin University, Perth, WA Australia; Himachal Pradesh Govt. Dental College & Hospital (IGMC), Shimla, India; Department of Psychology, St. Bede’s College, Himachal Pradesh University, Shimla, India; Menzies Health Institute Queensland and School of Dentistry and Oral Health, Griffith University, Gold Coast, QLD Australia; Institute for Positive Psychology and Education, Australian Catholic University, Sydney, Australia

**Keywords:** Dental cleaning, Flossing, Motivation, Self-efficacy, Intention, Incentives

## Abstract

**Background:**

Periodontal disease is a significant public health issue worldwide. Motivational techniques in combination with financial incentives are shown to lead to effective behavior change. The current study sought to examine whether a brief oral health promotion program (self-management cues that were based on self-efficacy and self-regulatory skills) in combination with an incentive (free dental treatment) would make a difference in the adoption of regular dental flossing in a population of Indian periodontal disease outpatients.

**Methods:**

One hundred and twelve participants (*n* = 55 oral health promotion intervention group; *n* = 57 control group) were assigned to the intervention (self-management cues + incentive) or control groups, and follow-up assessments were performed three weeks later. Flossing frequency, behavioral intentions, and perceived self-efficacy served as dependent variables. Data were analyzed with mixed models, ANCOVAs, and path analyses.

**Results:**

The intervention yielded effects on flossing frequency (*p* < 0.01) and flossing intentions (*p* < 0.01) at follow-up. Women developed stronger intentions than men. Moreover, by path analysis a sequential mediation chain was found that demonstrated an indirect effect of the intervention on flossing via self-efficacy and intentions: the intervention predicted changes in self-efficacy which, in turn, were associated with changes in intentions, predicting flossing frequency at follow up, while controlling for baseline behavior, gender, and age.

**Conclusions:**

Combining incentives with minimal self-management cues has been found effective in improving interdental cleaning intentions and habits in periodontal disease patients, and the facilitating role of dental self-efficacy has been demonstrated.

## Background

The World Health Organization's (WHO) World Oral Health Report 2003 states that dental caries affects 60–90 % of schoolchildren and the vast majority of adults [1]. Similarly, the WHO databank on periodontal disease reports 10 to 15 % of adult populations suffering from the most severe forms of the disease, whereas gingival bleeding and calculus are the most prevalent [2]. Dental caries and periodontal disease are major causes of tooth loss, which then impacts on people’s quality of life in terms of functionality, self-esteem, and social relationships [2]. Both are attributed to poor oral hygiene, with non-compliance to protective measures and patient behavior leading to unnecessary diagnostic and treatment procedures thus resulting in substantial social, health, and economic costs [3].

Interdental cleaning is an effective preventive measure which will impact on both dental caries and periodontal disease. Interdental cleaning is the practice of removing trapped food between the teeth and the biofilm of bacteria (dental plaque) that forms around the teeth and gums. Traditionally, dental floss has been used to achieve this and a systematic review concluded that flossing, in addition to toothbrushing, reduces gingivitis compared to toothbrushing alone [4]. Another systematic review on the effect of interdental brushing on oral diseases in adults reported the beneficial effects of interdental brushing reducing gingivitis, but insufficient evidence is available to determine whether interdental brushing had any benefit towards dental plaque when compared to flossing [5]. Furthermore, regular dental flossing is an effective adjunct to toothbrushing as its benefits outweigh any potential harm in avoiding plaque formation [4].

Regular interdental cleaning, such as daily dental floss use, is an uncommon behavior [6], practiced by few individuals worldwide including in India [7], resulting in a large proportion of people who floss their teeth less than the recommended time or not at all. Given that both dental caries and periodontal disease are largely preventable, it is likely that decisions informing individuals’ behavior to prevent these oral diseases have psychological origins. Prior research has shown that lack of self-efficacy and self-regulatory skills are associated with a disinclination to change dental flossing behavior [8, 9]. Raising people’s self-efficacy and providing them with sufficient skills (such as setting goals) is likely to increase their motivation translating into action. Thus, adopting such a strategy may be effective in improving people’s flossing habits. In addition, a number of countries (see e.g., [10]) have adopted healthcare policies providing individuals with financial assistance for their dental care needs with the aim to promote good oral hygiene habits. Previous research has found financial incentives to facilitate behavior change [11, 12]. Furthermore, meta-analytic research has found motivation and extrinsic incentives to jointly predict behavior, suggesting that the two are not necessarily antagonistic (in that incentives erode motivation) but should rather be considered simultaneously [13].

### Motivation and self-regulation toward interdental cleaning

A close examination of the major motivational theories that have been applied to the understanding of health behavior assume that a motivation to act or intention formation (i.e., the amount of effort one invests in order to pursue an action) is the most proximal predictor of behavior. In the initial stage of health behavior change, people need to develop a motivation [14]. Psychological constructs such as self-efficacy and self-regulation often serve as a theoretical backdrop to motivation formation [15, 16]. Perceived self-efficacy is the confidence in one’s ability to execute a difficult or resource-demanding behavior [17]. The difficulty here is not a technical demand of interdental cleaning but rather its regular performance as an integrated part of people’s daily life. Self-efficacy has been shown to predict a wide range of health behaviors including oral self-care [18, 19]. Moreover, to adopt or maintain regular interdental cleaning, one can be motivated by self-efficacy followed by self-regulatory skills, such as planning and action control, to translate motivation into actual dental cleaning performance [8, 15, 20]. Studies have reported beneficial effects of such self-regulatory skills on dental flossing [21, 22], and a combination of self-efficacy and planning has been found to be associated with higher frequency in performing dental self-care [23]. In a cluster randomized controlled trial with Iranian adolescent girls, Gholami et al. [24] identified positive effects of a brief self-regulatory intervention on dental flossing, in which changes in self-efficacy mediated between treatment conditions and outcomes.

### Incentives to promote interdental cleaning

Behavioral incentives are motivating rewards, including anything provided by an external agent contingent on performance of target health behaviors (e.g., free or subsidized costs for specialized health services, awards, healthcare benefits and recognitions) [25]. Incentives demonstrate to people that they are viewed as worthy of being helped, and work particularly well when targeting groups who need extra support to remove some of the financial barriers faced in trying to change health behaviors [11]. A systematic review found that financial incentives, in particular, were 1.2 to 2.5 times more effective for promoting behaviors than no intervention or usual care [11]. The acceptability of such incentives, however, rests on the incentive being fair to all recipients and members of the public and is given as a voucher rather than as cash [12]. To conclude, financial incentives are shown to have beneficial effects on people’s behavior change. Moreover, considering techniques to increase motivation in addition to providing incentives as part of a behavior change program is suggested to provide most effective results [12, 13].

### The aim of the current study

The current study investigates an educational oral health promotion program to improve motivation and interdental cleaning habits among periodontal disease outpatients. The program consisted of two components: a worksheet with self-management cues and free dental treatment as an incentive. To evaluate the effectiveness of the program in terms of changes in behavioral intention and dental flossing, a passive control group was randomly selected. It was expected that participants in the health promotion intervention group compared to the control group would attain higher scores in intention (Hypothesis 1) and behavior (Hypothesis 2) at the follow-up assessment three weeks later. In addition, self-efficacy, as the putative active ingredient, was expected to be higher at follow-up in the health promotion group compared to the control group (Hypothesis 3); and that self-efficacy and intention would serve as mediators between intervention conditions and flossing at follow-up (Hypothesis 4).

## Method

### Participants and procedure

The study adopted an experimental 2 (condition) × 2 (time) research design with a three week follow-up. The study received ethical approval by the hospital’s internal review board. Study participants were recruited between October and December, 2014 during regular outpatient visits at the Dental College and Hospital, Shimla, Himachal Pradesh, India. A blinded research associate (third author) invited the outpatients to participate voluntarily in a study on preventive oral hygiene. To participate, patients needed to be 18 years of age or older and clinically diagnosed with having periodontal disease. Informed consent was obtained prior to participation, and anonymity was assured by the use of an identification code. The research team had no access to patients’ health records; however, the dentists provided general information about their patient sample without disclosing individual details. From this qualitative information, it was observed that patients had a broad range of diagnoses, from mild gingivitis to severe aggressive periodontal disease, and about 10 % had oral cancer without being aware of it. Many participants were illiterate and reported having pain in the teeth or mouth. Some of the participants reported engaging in self-treatment using home crude remedies such as salt and lemon mixed with masala (herbs) to heal the inflammation and minimize the pain.

**Fig. 1:**
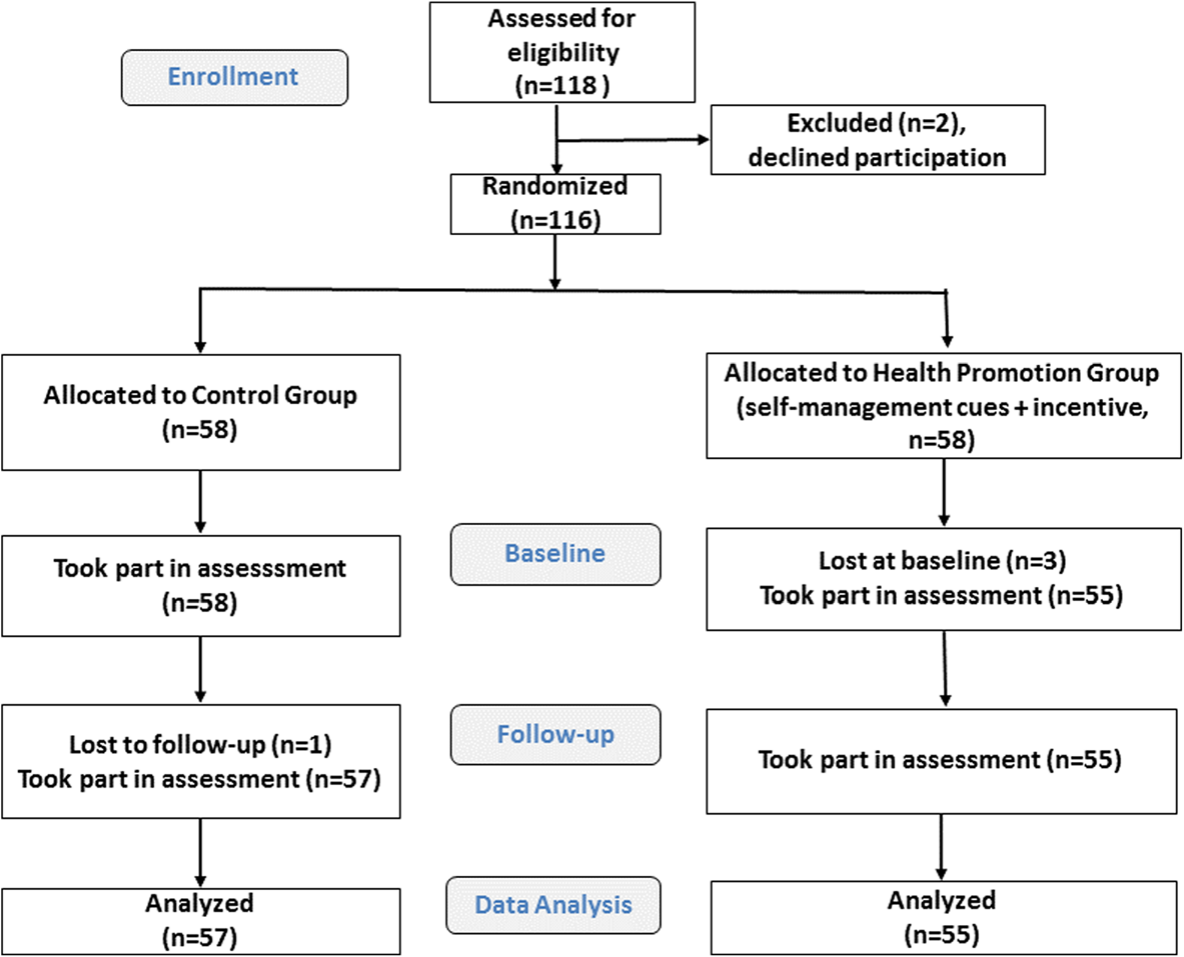
Flow diagram outlining participant allocation into the dental flossing health promotion group or the control group

One hundred and eighteen participants were assessed for eligibility of which two declined to participate. A total of 116 patients were recruited and allocated to either an intervention (*n* = 58) or a control condition (*n* = 58) by cluster randomization. Participants were blinded about the allocation throughout the study. Three patients from the oral health promotion intervention group were lost at baseline; thus, a total of 113 patients (67 % women; Mean age = 27.05 years, SD = 12.75, ranging from 18 to 69 years) participated in the study. Three weeks later, patients were re-invited to complete the follow-up questionnaire. One participant from the control group was lost at follow-up. Final analyses, therefore, were based on 112 participants (*n* = 55 health promotion intervention group; *n* = 57 control group). See Fig. 1.

### Intervention content

Only the intervention group received the intervention package after baseline measurement. The intervention consisted of two components: a brief psychological component of self-management cues and an incentive-based component. The self-management cues comprised strategies targeting self-efficacy and self-regulatory skills (see [26]) and consisted of a two-page leaflet that included information about oral hygiene (i.e., what it is, why it is done, how it is done, and the health consequences), along with a goal setting exercise (planning when, where, and how to floss) and instructions on how to practice oral self-care. The purpose was to explore the feasibility of a very brief intervention following an idea by Sniehotta et al. [22] who conducted a one-minute intervention for changing oral self-care behavior. The incentive component comprised a financial dental care assistance incentive in the form of a free dental treatment including checkups, dentures, removing of caries, fillings, and dental aids free of charge during the study period. This was made available from a scheme called Muskan Yojna, launched by the Department of Public Health Dentistry with the purpose of giving easy oral care access to patients below the poverty line. Participants in the control group received no intervention; neither free dental aids nor instructions on what, why, and how to perform their oral self-care. They were allocated to the study during their usual dentist visits for which they cover the cost themselves, and responded only to the questionnaires at the two assessment points.

### Measures

#### Behavior

Dental flossing behavior was assessed at baseline (Time 1) and follow-up (Time 2). Participants were asked to indicate the number of times they had flossed their teeth in the previous week; “During the last week, I have flossed my teeth _____ times per day”.

#### Intention

Intention was measured at Time 1 and Time 2 with the stem item: ‘How often do you intend to floss your teeth per day?”, followed by responses ranging from *do not intend to floss at all* (0), *intend to floss once per day* (1), *intend to floss twice per day* (2) to, *intend to floss three times per day* (3)”.

#### Self-efficacy

Flossing self-efficacy was assessed with two items at Time 1 (Spearman’s ρ = .88, Cronbach’s alpha = .87) and Time 2 (Spearman’s ρ = .83, Cronbach’s alpha = .83). The item stem “I am confident that I can floss my teeth this week on a regular basis…” was followed by the items “…even if it is time consuming” and “…even when it takes a long time to become part of my daily routine”. Responses were scored on a four-point Likert scale ranging from *not at all true* [1] to *exactly true* [4]. The two items were averaged to form the flossing self-efficacy scale.

### Analytical procedure

Using SPSS 23, independent-sample *t*-tests, *χ*^2^ test and MANOVA were used for attrition analysis. Intervention effects on changes in flossing as well as flossing intentions are tested with the SPSS MIXED procedure using linear 2-level models with time points nested in individuals. Flossing as well as flossing intentions are level-1 dependent variables, whereas intervention conditions serve as a between-subjects covariate (level-2), and time as a within-subjects factor. The group by time interaction (cross-level interaction) will be the main test of the primary hypotheses. In a linear mixed-effects model, the responses from participants (e.g., flossing rates) are thought to be the sum of fixed and random effects. Random effects contribute only to the covariance structure of the data. The fixed effects are of primary interest, but adjustment for the covariance structure makes the results more accurate [27]. Moreover, univariate analyses of covariance (ANCOVA) are computed with Time 2 flossing as well as flossing intentions as dependent variables, intervention conditions and gender as between-subjects factors, and baseline scores as well as age as covariates. Based on a power of 0.80 (*p* = 0.05) and an effect size of 0.12, a sample size of *N* = 112 was appropriate for this kind of analysis.

A sequential mediation model was conducted by means of the SPSS Process macro Hayes [28]. Intervention conditions were specified as the most distal antecedent, dental self-efficacy served as the first mediator whereas the behavioral intention served as the second mediator in a row, which also constituted the most proximal predictor of Time 2 dental flossing. This sequential model was extended by inclusion of three covariates to control for individual differences in baseline flossing, age, and gender. Confidence intervals (95 %) were generated by bootstrapping with 5,000 re-samples. Bootstrapping is a non-parametric re-sampling procedure that allows generating confidence intervals for statistical inference where normality assumptions about the sample distribution are not required. It is recommended for mediation analyses, including serial multiple mediation models [28]. The entire analysis was then replicated by structural equation modeling using AMOS 21 with full information maximum likelihood (FIML). The latter procedure provided the standardized parameter estimates (betas).

## Results

### Preliminary analyses

Demographic details of the study participants along with the means, standard deviations, and group comparison statistics are summarized in Table 1. Correlations between the study variables are summarized in Table 2. Age differences occurred (*p* < 0.01), with greater numbers of older patients assigned to the health promotion intervention group (*M* = 31.58, *SD* = 14.05) than to the control group (*M* = 22.76, *SD* = 9.68). No gender differences between groups were found (*p* > 0.05).

**Table 1:** Means and standard deviations (SD) of study variables and pairwise comparisons between the two groups at two measurement points in time

		Control group (*n* = 57)	Intervention group (*n* = 55)		Effect size
Variables	Time points	Means (*SD*)		*p*	*eta* ^*2*^
Flossing	Baseline	1.00 (0.80)	1.11 (0.74)	0.46	0.01
–	Follow-up	1.02 (1.01)	1.89 (0.90)	<0.001	0.18
Self-efficacy	Baseline	2.03 (0.92)	1.64 (0.75)	0.02	0.05
–	Follow-up	2.45 (0.95)	2.63 (0.99)	0.35	0.01
Intention	Baseline	0.96 (0.94)	1.16 (0.66)	0.20	0.02
–	Follow-up	1.33 (1.30)	2.09 (0.87)	<0.001	0.11
Age	–	22.81 (9.76)	31.58 (14.05)	<0.001	0.12
Gender (Female/Male)	–	42/16	34/21	0.26	0.01

**Table 2:** Pearson correlations of dental flossing, intention, self-efficacy, age, and gender

	Variables	2	3	4	5	6	7	8
1.	Flossing T1	.21*	.63**	.37**	.10	.14	.12	.25**
2.	Self-efficacy T1		.17	.01	.26**	−.06	−.08	−.03
3.	Flossing Intention T1			.27**	.06	.26**	.07	.21**
4.	Flossing T2				.34**	.59**	.21*	.08
5.	Self-efficacy T2					.50**	.00	.08
6.	Intention T2						.14	.15
7.	Age							.29**
8.	Gender							

*T1* Time 1, *T2* Time 2

*p < 0.05; **p < 0.01

### Intervention effects

#### Dental flossing

Two-level linear mixed models were computed with time points nested in individuals, using flossing frequencies at both time points as the level-1 dependent variable and intervention conditions (groups) as well as gender as level-2 covariates. The results revealed neither a main effect of gender nor an interaction of gender and time. Therefore, we report the corresponding analyses without the covariate gender (see Table 3). The intercept of 1.02 describes the ending status of the control group (flossing, Time 2). The group estimate of 0.87 (*p* < 0.01) reflects the difference to the treatment group which means that 1.02 + 0.87 = 1.89 is the Time 2 mean for the treatment group. The time estimate of −0.02 reflects the initial status of the control group (1.02−0.02 = 1.00 at Time 1). The cross-level interaction estimate indicates that there was a steep increase for the treatment group over time (0.76, *p* < 0.01). The estimates of covariance parameters signify no variance at Time 1 and a large variance at Time 2 (*p* = 0.01). Also, the variance component of 0.28 (*p* < 0.01) at the person level (level 2) is significant.

**Table 3:** Estimates of linear mixed model over 20 days for flossing and flossing Intentions as a function of intervention (*N* =112)

Model parameters for flossing				95 % CI	
Fixed effects (intercept, slopes)	Estimate (SE)	*t*	*p*	Lower Bound	Upper Bound
Intercept	1.02 (0.13)	8.04	<0.01	0.08	1.27
Group	0.87 (0.18)	4.84	<0.01	0.52	1.23
Time	−0.02 (0.13)	−0.14	0.89	−0.27	0.24
Group x Time	−0.76 (0.18)	−4.15	<0.01	−1.13	−0.40
Estimates of covariance parameters for flossing		Wald’s z			
Repeated Measures Var1	0.04 (0.13)	0.28	0.78	0.00	38.06
Repeated measures Var2	0.35 (0.14)	2.56	0.01	0.17	0.76
Intercept + time (subjects)	0.28 (0.08)	3.71	<0.01	0.16	0.47
Model parameters for intentions				95 % CI	
Fixed effects (intercept, slopes)	Estimate (SE)	*t*	*p*	Lower Bound	Upper Bound
Intercept	1.12 (0.16)	7.09	<0.01	0.80	1.43
Group	1.04 (0.22)	4.70	<0.01	0.60	1.47
Time	−0.38 (0.18)	−2.19	0.03	−0.73	−0.04
Gender	0.77 (0.26)	2.98	0.003	0.26	1.28
Group x Time	−0.56 (0.23)	−2.44	0.02	−1.02	−0.11
Group x Gender	−0.93 (0.28)	−3.37	<0.01	−1.48	−0.38
Time x Gender	0.06 (0.25)	0.23	0.82	−0.43	0.54
Estimates of covariance parameters for intentions		Wald’s z			
Repeated Measures Var1	0.36 (0.17)	2.16	0.03	0.15	0.90
Repeated measures Var2	0.84 (0.20)	4.28	<0.01	0.53	1.33
Intercept + time (subjects)	0.14 (0.08)	1.68	0.09	0.04	0.44

All *p* values are two-tailed except in the case of variances, where one-tailed *p*-values are used (because variances are constrained to be non-negative). Time is coded 0 = baseline, 1 = follow-up. Group is coded 0 for the control group and 1 for the treatment group

#### Flossing intentions

The same type of 2-level linear mixed models were computed for flossing intentions as dependent variable, including gender as an additional level-2 covariate because preliminary analyses revealed significant gender effects (see Table 3). The intercept of 1.12 describes the ending status of the control group (flossing intentions, Time 2). The group estimate of 1.04 (*p* < 0.01) reflects the difference to the treatment group which means that 1.12 + 1.04 = 2.16 is the Time 2 mean for the treatment group. The time estimate of −0.38 reflects the initial status of the control group (1.12−0.38 = 0.74 at Time 1). The cross-level interaction estimate indicates that there was a steeper increase for the treatment group over time (0.56, *p* = 0.02). The gender estimate of 0.77 (*p* < 0.01) indicates that men (coded 1) had a follow-up mean score of 1.89 (independent of intervention conditions). The group x gender estimate of −0.93 (*p* < 0.01) signifies the difference between intervention conditions for women as opposed to men. The time x gender interaction, on the other hand, was not significant. The covariance parameter estimates signify substantial variance at both time points whereas the person level (level 2) variance component is no longer significant due to the inclusion of level-2 covariates.

#### Gender differences

**Fig. 2:**
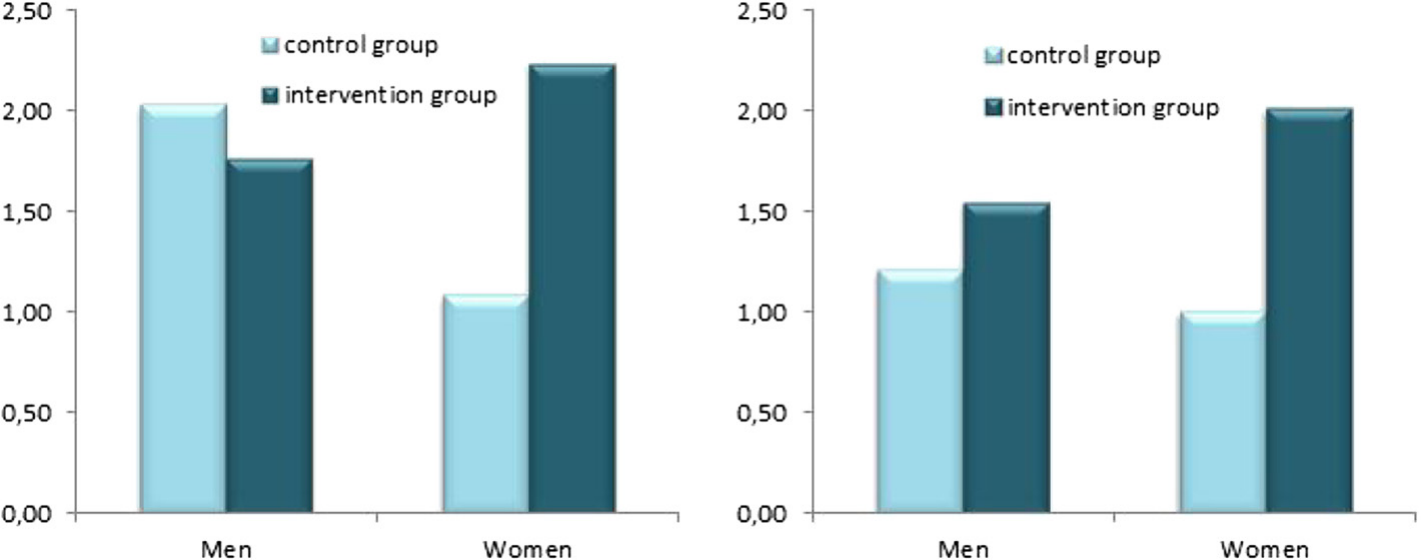
Follow-up means of dental flossing intentions (*left panel*) and dental flossing frequency (*right panel*) adjusted for baseline levels and age

ANCOVA results implied that women benefitted more from the intervention than men resulting in higher levels in flossing frequency and flossing intentions (see Fig. 2). Using univariate analysis of covariance (ANCOVA) with baseline (Time 1) flossing, age, and gender as covariates, it was found that there were group differences for flossing at follow-up (Time 2), *F*(1, 106) = 12.77, *p* = 0.001, η^2^ = 0.11. The intervention group obtained a higher level of flossing frequency, *M* = 1.79, *SE* = .13, 95 % CI [1.53; 2.04] than the control group, *M* = 1.11, *SE* = .13, 95 % CI [0.85; 1.37]. This finding confirms the Hypothesis 1. Group means, adjusted for the covariates, are displayed in Fig. 2 (right panel). For flossing intentions, ANCOVA with the same covariates also yielded group differences at follow-up (Time 2), *F*(1, 106) = 3.94, *p* = 0.05, η^2^ = 0.04. The intervention group obtained higher levels of flossing intentions, *M* = 2.01, *SE* = .15, 95 % CI [1.71; 2.31] than the patients in control group, *M* = 1.57, *SE* = .16, 95 % CI [1.26; 1.88] confirming Hypothesis 2. Group means, adjusted for the covariates, are displayed in Fig. 2 (left panel).

### Testing the mechanisms: a sequential mediation chain

**Fig. 3:**
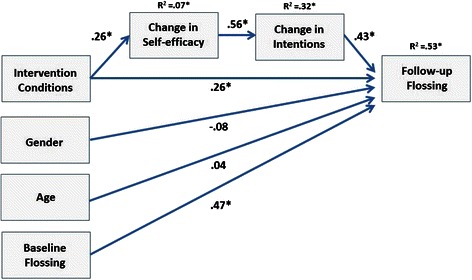
Mediation chain predicting dental flossing by treatment via changes in self-efficacy and intentions, controlling for baseline flossing, gender, and age. Full information maximum likelihood estimates, *N* = 112. Note: Baseline intercorrelations omitted for easier communication. Gender (1 = male, 0 = female), intervention conditions (1 = treatment, 0 = controls), * = *p* < 0.01

First, testing the model with manifest variable regressions revealed that there remained a direct effect between conditions and Time 2 flossing, *p* < 0.01, CI 95 % [.25, .90]. Also, the covariates age, *p* = 0.58, CI 95 % [−.01, .02] and gender, *p* = 0.09, CI 95 % [−.65, .05] had no relationship to the target variable. The sequential mediation chain via two mediators yielded an indirect effect, *p* < 0.05, CI 95 % [.01, .23] whereas the other pathways did not yield significant indirect effects. Second, structural equation model fit was *χ*2 (8 df) = 14.4, *p* = 0.07, *χ*2/df = 1.8, CFI = .95, RMSEA = 0.08 [.0, .15]. Figure 3 displays all standardized full information maximum likelihood estimates based on structural equation modeling with AMOS. Of the flossing variance, 53 % were accounted for by baseline flossing (ß = .47), intervention conditions (ß = .26), and intentions (ß = .43). Group membership predicted changes in self-efficacy (ß = .26), although there was no significant effect on Time 2 self-efficacy, as found in the previous analyses. Changes in self-efficacy predicted changes in intentions (ß = .56). Control variables age and gender did not contribute to the prediction. Three mediation pathways were tested by bootstrapping: (a) the sequential mediation chain via two mediators yielded an indirect effect, β = .10, *p* < 0.05, CI 95 % [.01, .23]; (b) the simple mediation path from group via self-efficacy to flossing was not significant; and (c) the simple mediation from group via intention to flossing was also not significant.

## Discussion

Previous research has found motivational techniques in combination with financial incentives lead to effective behavior change [13]; however, further research is needed to confirm such suggestions. Given periodontal disease is a significant public health issue worldwide [1], the current study sought to examine whether a brief health education promotion program (worksheet with self-management cues that were based on self-efficacy and self-regulatory skills) in combination with an incentive (free dental treatment) would make a difference in the adoption of regular dental flossing in a population of Indian periodontal disease outpatients. Changes in motivation (defined as behavioral intention) and flossing as outcomes were assessed three weeks after baseline.

In the current study, the time x group interaction for self-efficacy did not reach significance, disconfirming Hypothesis 3. However, a significant interaction was found for intention and flossing in which the intervention group improved in terms of these two outcome variables, confirming Hypothesis 1 and 2. Moreover, self-efficacy and intention were specified in a path model as mediators between intervention conditions and subsequent dental flossing behaviours, confirming Hypothesis 4. The findings revealed a sequential mediator model in which first changes in self-efficacy and afterwards changes in intention mediated between intervention conditions and behavioral outcomes. In their Iranian sample, Gholami et al. [24] identified a similar sequential mediation via intention and self-efficacy on dental flossing. In their study, however, the two mediators were placed in a different order, suggesting first changes in intentions and afterwards changes in self-efficacy mediated between intervention conditions and behavioral outcomes. This difference in ordering may be the result of the type of self-efficacy construct examined and its item wording used in different studies. Self-efficacy as a stage-specific construct can be a predictor of intention in an earlier stage of health behavior change (as in the current study), or it can be a most proximal predictor of behavior at a later stage of change (as in Gholami et al’s study). Nevertheless, the sequential mediation chain identified in these studies highlight the fact that both self-efficacy and intention play a significant role in the mechanism that facilitates dental flossing. Results indicate the mediating role of behavioral intention and self-beliefs in predicting the desired health behavior (dental flossing) in periodontal disease patients. Thus, the findings illustrate that oral awareness promotes the formation of behavioral intentions as well as stronger self-beliefs (self-efficacy) for increased oral health behaviors which, in turn, were associated with better oral health status in dental patients as found in previous studies [3, 18]. Such findings are in line with studies documenting that intention and self-efficacy serve as proximal predictors of dental flossing, and often as mediators (e.g., [21, 29, 30]). *The current study may contribute to develop interventions facilitating dental self-care to improve oral health status* (see review [31]). Finally, although not an explicit research question of the current study, a gender effect on flossing intentions as well as a group x gender interaction was observed in that females benefitted more than males from the intervention. This finding is in line with other research suggesting gender differences are evident for health behaviors [16], including for oral hygiene care behaviors [32, 33].

The current study suffers from some limitations. Assessments were self-reported and dental flossing was measured retrospectively. One could use on-going behavioral assessments such as dental calendars that individuals can deposit in their bathrooms to tick every flossing incident [8]. In addition, flossing intention was assessed by a single item which limits assessing its reliability. Single item measures, however, are in line with a large number of health psychology studies (e.g., [34]). Furthermore, the oral health promotion program consisted of a multi-component approach involving self-management cues with an incentive and, as such, cannot disentangle the most active ingredient. Finally, periodontal disease patients need daily interdental cleaning for infection control; thus, the short term follow-up period in the current study needs to be extended to determine the longer-term effects of the program.

## Conclusion

Nevertheless, the current study’s brief oral health promotion intervention yielded positive effects on dental flossing intentions and behavior in a group already diagnosed with periodontal disease. Moreover, the current study was able to elucidate the mechanisms of changing dental flossing behaviors in a group at risk for further oral disease issues. The findings partly replicate similar studies [20, 24, 33] and, thus, make a contribution to the cumulative knowledge about psychological components in dental hygiene behavior change.

## Abbreviations

ANCOVA: analysis of covariance
CFI: comparative fit index
CI: confidence interval
MANOVA: multivariate analysis of variance
RMSEA: root mean square error of approximation
